# Bioinformatics Study of Cancer-Related Mutations within p53 Phosphorylation Site Motifs

**DOI:** 10.3390/ijms150813275

**Published:** 2014-07-29

**Authors:** Xiaona Ji, Qiang Huang, Long Yu, Ruth Nussinov, Buyong Ma

**Affiliations:** 1 State Key Laboratory of Genetic Engineering, School of Life Sciences, Fudan University, Shanghai 200433, China; E-Mails: xiaonaji@163.com (X.J.); huangqiang@fudan.edu.cn (Q.H.); 2 Basic Science Program, Leidos Biomedical Research, Inc., Cancer and Inflammation Program, National Cancer Institute, Frederick, MD 21702, USA; E-Mail: nussinor@helix.nih.gov; 3 Sackler Institute of Molecular Medicine, Department of Human Genetics and Molecular Medicine, Sackler School of Medicine, Tel Aviv University, Tel Aviv 69978, Israel

**Keywords:** phosphorylation, p53 protein, p63, p73, protein binding site, cancer, intrinsically disordered proteins

## Abstract

p53 protein has about thirty phosphorylation sites located at the *N*- and *C*-termini and in the core domain. The phosphorylation sites are relatively less mutated than other residues in p53. To understand why and how p53 phosphorylation sites are rarely mutated in human cancer, using a bioinformatics approaches, we examined the phosphorylation site and its nearby flanking residues, focusing on the consensus phosphorylation motif pattern, amino-acid correlations within the phosphorylation motifs, the propensity of structural disorder of the phosphorylation motifs, and cancer mutations observed within the phosphorylation motifs. Many p53 phosphorylation sites are targets for several kinases. The phosphorylation sites match 17 consensus sequence motifs out of the 29 classified. In addition to proline, which is common in kinase specificity-determining sites, we found high propensity of acidic residues to be adjacent to phosphorylation sites. Analysis of human cancer mutations in the phosphorylation motifs revealed that motifs with adjacent acidic residues generally have fewer mutations, in contrast to phosphorylation sites near proline residues. p53 phosphorylation motifs are mostly disordered. However, human cancer mutations within phosphorylation motifs tend to decrease the disorder propensity. Our results suggest that combination of acidic residues Asp and Glu with phosphorylation sites provide charge redundancy which may safe guard against loss-of-function mutations, and that the natively disordered nature of p53 phosphorylation motifs may help reduce mutational damage. Our results further suggest that engineering acidic amino acids adjacent to potential phosphorylation sites could be a *p53* gene therapy strategy.

## 1. Introduction

p53 plays a central role in controlling cellular life and death by integrating many pathways related to apoptosis, cell arrest, and DNA repair, in response to various types of stress [1,2]. In addition to its critical role as a tumor suppressor, it regulates hundreds genes and is a guardian maintaining genome stability [1,3]. Two other p53 protein families, p63 and p73, have high level structural and functional similarities with p53, especially in transactivating similar genes and maintaining similar interaction networks [4]. However, p53, p63 and p73 have different biological tasks. Aberrancies in all p53 protein families are strongly implicated in cancer progression and metastasis [5].

p53 phosphorylation [6,7] at almost 30 Ser/Thr residues regulate its stability and activity [8,9], localization [10,11], and tetramerization [12,13]. Phosphorylation of S15, T18, and S20 has been shown to strongly disrupt p53–MDM2 binding and trigger aberrant consequences [14,15]. In DNA damage, S15 phosphorylation promotes repair of DNA breaks [16]. S15 phosphorylation may also be coupled with T55 dephosphorylation [17]. Phosphorylation of S46 may induce p53-regulated apoptosis inducing protein 1) (p53AIP-1) expression and apoptotic response to DNA damage [18,19]. However, in minor DNA damage, p53 represses its own phosphorylation at S46 [20]. T55 phosphorylation exports p53 from a nuclear to a cytoplasmic localization [21]. Phosphorylation of S149, T150 and T155 lead to degradation of p53 through the COP9 (constitutive phofomorphogenesis 9) pathway [22,23]. Phosphorylation of S315 enhances nuclear retention of p53 [24]. Phosphorylation of S315 and S376 may induce p53 degradation in endoplasmic reticulum (ER) stressed cells [25] or MDM2 mediated inhibition of p53 [26].

Given the functions controlled by p53 phosphorylation, it is expected that the specificity and dynamics of p53 phosphorylation are carefully regulated, and mutations would affect p53 phosphorylation pattern and function. Phosphorylated p53 mutants were found to accumulate in tumor tissues [27,28]. p53 has at least 167 mutations that are phosphorylation-related and they are involved in many types of cancers [29]. For example, phosphorylation at S15 and S392 in mutant p53 molecules differs from wild-type p53 [30]. It was proposed that phosphorylation of mutant proteins at S15 and S315 is related to gain-of-function mutants in DNA homologous recombination [31]. AURKA–TP53 signaling through phosphorylation of S215 can be disrupted by six direct and seven flanking phosphorylation-related point mutations, leading to increased activity of p53 [32]. Several reports indicated that phosphorylation and mutations are coupled in p53 functional changes [33,34,35,36]. It has been observed that p53 phosphorylation can have long range allosteric effects [37,38,39], and mutations away from phosphorylation sites can also be coupled and alter p53 function [27,30,40].

Unlike phosphorylation of p53, the function of Ser/Thr phosphorylation for p73 was not known previously [41,42], although Tyr phosphorylation has been investigated [43]. Accumulation of p73 after DNA damage is primarily mediated by the tyrosine kinase c-Abl [44]. Following a genotoxic insult such as γ-irradiation or cisplatin treatment, p73 interacts with c-Abl via its PxxP motif at the *C*-terminal homo-oligomerization domain and becomes phosphorylated predominantly at Y99, and also at Y121 and Y240 [45] The consequences of the Ser/Thr phosphorylation in p73 were studied [46]. Phosphorylation of T27 in p73 can regulate its transactivation [47,48]. Several Ser/Thr residues are now known to be phosphorylated by c-Jun *N*-terminal kinase [49,50]. Burge *et al.* found that the *N*-terminal region of p73 is similar to that of p53, with phosphorylation of T14 modulating the p73N-p300 interaction and transactivation [51]. In the *C*-terminal of p73, the phosphorylation of S388 by protein kinase C is important in cell-cycle regulation [52]. To date, it has been established that p73 S47, T86, T167, S289, S388, T422, and T482 can be phosphorylated [53]. p63 was also found to be phosphorylated [5,54,55] in UV radiation [56] and IR-triggered [57] responses. High level of p63 phosphorylation is involved during wound healing [58]. Phosphorylation of T397/S383 controls p63-Dlx3 interaction and p63 degradation [59,60].

p53-targeted therapy has been increasingly considered in cancer treatment [61], and many of the p53-targeted approaches are related to p53 phosphorylation [62,63,64]. For example, Akt promotes cisplatin resistance in human ovarian cancer cells through inhibition of p53 phosphorylation and its repressor function in the nucleus [65]. However, Luteolin sensitizes the anticancer effect of cisplatin by supporting the c-Jun NH_2_-terminal kinase-mediated p53 phosphorylation and stabilization [66], which underscores the importance of understanding cancer-related mutations within p53 phosphorylation site motifs.

Here we study p53 phosphorylation through using bioinformatics approaches to investigate sequence patterns and mutations within p53 phosphorylation site motifs, including the flanking residues. We first identify the mutation pattern in the phosphorylation motifs, and then analyze the amino acid correlations at sites within the phosphorylation motifs to identify common and unique amino acids correlations across p53/p63/p73. We also computationally analyzed the disorder propensity of the phosphorylation motifs and found that human cancer mutations within phosphorylation motifs tend to decrease the disorder propensity. The unique features of p53’s phosphorylation motifs reflect an evolutionary pressure to selectively bind to a specific kinase in response to phosphorylation signals.

## 2. Results

### 2.1. Sequence Features of Phosphorylation Sites in p53

#### 2.1.1. Consensus Motifs within the Phosphorylation Sites in p53

p53 has four domains (TAD1, TAD2, Core domain, and *C*-terminal domain) with numerous phosphorylation sites [6,7,9,67] (Table 1). The TAD1 domain contains S6, S9, T18, S15, S20, S33, and S37. This group is within the p53–MDM2 binding site and is also important for interactions with CBP/p300 proteins. The TAD2 and nearby proline rich segments contain S46, T55, T81, S99 and S106. The core domain has the phosphorylation sites of S149, T150, and T155 leading to the degradation of p53 through the COP9 pathway. Recently, Gully *et al.* found that Aurora B kinase phosphorylates p53 at the core domain S183, T211, and S215 to also induce p53 degradation [68]. In the *C*-terminal domain, there are S313, S315, S376, T377, S378, T387, and S392, which enhance p53-DNA interaction and transactivation upon phosphorylation. Phosphorylation of S362/S366 was also reported [69]. In an extreme case, one study suggested that almost all Ser and Thr residues in p53 can be phosphorylated [70].

**Table 1:** Phosphorylation site motifs for p53.

P_−3_	P_−2_	P_−1_	pS/pT	P_+1_	P_+2_	P_+3_	Kinases	Consensus Motif	Ref.
E	P	Q	S6	D	P	S	JNK2, CK1δ	P-X-S/T-P	[6,7]
S	D	P	S9	V	E	P	CK1ε	pS-X-X-S/T	[6,7]
P	P	L	S15	Q	E	T	CDK5, mTOR, ATM	P-L-S/T-P (CDK) D-S/T-Q-E (ATM)D	[14,15]
S	Q	E	T18	F	S	D	Chk2, TTK, VRK1		[14,15]
E	T	F	S20	D	L	W	Chk2, Plk3	T-X-S/T-X-X-W (Chk2)	[14,15]
N	V	L	S33	P	L	P	Cdk5/7/9, GSK3β, p38K	P-L-S/T-P (CDK)	[6,7]
P	L	P	S37	Q	A	M	ATR, PRAK	L-P-S/T-Q-A (ATR)	[6,7]
L	M	L	S46	P	D	D	Cdk5, p38K, PKC	P-L-S/T-P (CDK)	[20]
Q	W	F	T55	E	D	P	ERK2, TAF1		[21]
A	A	P	T81	P	A	A	JNK2	P-X-S/T-P	[6,7]
S	V	P	S99	Q	K	T	ATM, ATR	V-P-S/T-Q (ATR)	[6,7]
Y	Q	G	S106	Y	G	F	Aurora A		[6,7]
N	N	T	S313	S	S	P	Chk1/2		[6,7]
N	T	S	S314	S	P	Q	Chk1/2	T-X-S/T (Chk2)	[6,7]
T	S	S	S315	P	Q	P	STK15, Cdk9, CDK2	P-L-S/T-P (CDK)	[24,25]
P	G	G	S362	R	A	H	IKK2B		[6,7]
R	A	H	S366	S	H	L	IKK2, Chk2		[6,7]
K	G	Q	S376	T	S	R	PKC, GSK3β	R-K/R-X-S/T-X-K-K/R (PKC)	[25,26]
G	Q	S	T377	S	R	H	Chk1/2		[6,7]
Q	S	T	S378	R	H	K	Chk1/2, PKC	R-K/R-X-S/T-X-K-K/R (PKC)	[6,7]
M	F	K	T387	E	G	P	Chk1		[6,7]
G	P	D	S392	D			Cdk9, PKR, FACT		[30]
W	V	D	S149	T	P	P	CSN		[22,23]
V	D	S	T150	P	P	P	CSN		[22,23]
P	P	G	T155	R	V	R	CSN		[22,23]
E	R	C	S183	D	S	D	Aurora B	R/K-X-S/T (Aurara B)	[68]
D	R	N	T211	F	R	H	Aurora B	R/K-X-S/T (Aurara B)	[68]
F	R	H	S215	V	V	V	STK15, Aurora B	R/K-X-S/T (Aurara B)	[32]
G	R	N	S269	F	E	V	Aurora B	R/K-X-S/T (Aurara B)	[68]

Kinases usually recognize specifically a short peptide sequence containing the phosphorylation site (P-site) [71]. Mostly, the active site is large enough to interact with at least 7 substrate residues (with three amino acids on either side of the P-site). The residues in the *N*-terminal direction of the P-site are numbered P_−1_, P_−2_ and P_−3_, whereas these on the *C*-terminal side of the P-site are numbered P_+1_, P_+2_ and P_+3_. Thus a seven residue motif of P_−3_P_−2_P_−1_(S/T)P_+1_P_+2_P_+3_ may provide specific binding to protein kinases. Table 1 lists the seven residue motifs for the known phosphorylated sites. We probed the published work to check for the motifs to compare with known consensus substrate motifs of kinases. Proline occurs with high frequency, since many kinases require a proline right after the phosphorylation site to precisely align the substrate site to the catalytic pocket. One example is the CDK kinases, which phosphorylate many p53 sites, have strong preference for the SP motif [72]. S6 can be phosphorylated by JNK2, which has a MAPK consensus phosphorylation site of P-X-S/T-P [73]. The P-X-S/T-P roughly fits S6 (PQS6D). S9 fits the CK1 motif of pS-X-X-S/T, provided that S6 has been phosphorylated. S20 fits the Chk2 motif T-X-S/T-X-X-W [74]. ATR strongly recognizes the S/T-Q motif [75], and the S37 L-P-S/T-Q-A motif has a good representation in ATR consensus substrate sequences [75]. ATM kinase also recognizes the SQ motif, however, the ATM kinase can phosphorylate a non-S/T-Q motif S46 helped by docking dependent on the *N*- and *C*-terminal domains of p53 [39]. ERK2 has a consensus motif of P-X-S/T-P; however, it does not fit the T55 site. Aurora-A-mediated phosphorylation of p53 at S106 might inhibit its interaction with MDM2 [76]. However, this site does not fit the Aurora-A consensus motif R/K/N-R-X-S/T-B, where B denotes any hydrophobic residue [77], nor a looser K/R-ST-[not P] motif [78]. The only fit to Aurora-A is the absence of proline following the S/T site [77,78]. PKC usually requires distal docking sites for substrate specificity, with the pattern of R/K at positions −3, −2, +2, and +3 [79]. Thus, S376 may fit the PKC pattern in a broad sense. T150 and T155 sites might fit the general MAPK consensus phosphorylation site of P-X-S/T-P [73], however, these sites are specifically phosphorylated only by the COP9 signalosome (CSN) [22]. Overall, most phosphorylation motifs in the *N*-terminal region (nine out of 12) follow their consensus kinase substrate motif, while only four out of ten phosphorylation motifs in the *C*-terminal region fit the consensus kinase substrate motif pattern. The phosphorylation motifs in the core domain do not have known consensus kinase substrate pattern for S149, T150, and T155. However, S183, T211, S215, and S269 fit the Aurora-B pattern [68]. Overall, we are able to identify 17 consensus sequence motifs out of 29, reflecting kinase specificity as an important factor in p53 phosphorylation.

#### 2.1.2. Acidic Residues Occur at Positions P_−1_ and P_+1_ Adjacent to Phosphorylation Sites in p53

Surprisingly, acidic Asp/Glu appear at positions P_−1_ and P_+1_ (Table 2) with high rates. As can be seen in Table 2, Pro and Asp have the highest frequency, followed by Gln. Structurally, S/T-D/E-x-E/D is a phosphorylation site consensus sequence specifically recognized by casein kinase-2 [80]. In p53, the *C*-terminal VGPDS_392_D is such a Casein Kinase-2 site [80]. However, many other kinases known to phosphorylate p53 do not require this sequence combination [71]. Previously, we explored the dipeptide distributions of all X_i_Y_i+1_ pairs in p53 family proteins [77], and we observed that D/S dipeptides have the highest propensity in p53, while p63/p73 prefer Pro and Ser (P/S) dipeptides [77]. We re-examined previous data [77] and found that the high propensities of D/S and E/T are unique to p53. Both combine negatively charged residues adjacent to a potential phosphorylation site. Alternative combinations E/S and D/T are not preferred in p53, with ranking of E/S being 47 and D/T 161. For p63/p73, none of the four combinations (E/T, E/S, D/T, and D/S) is preferred.

**Figure 1:**
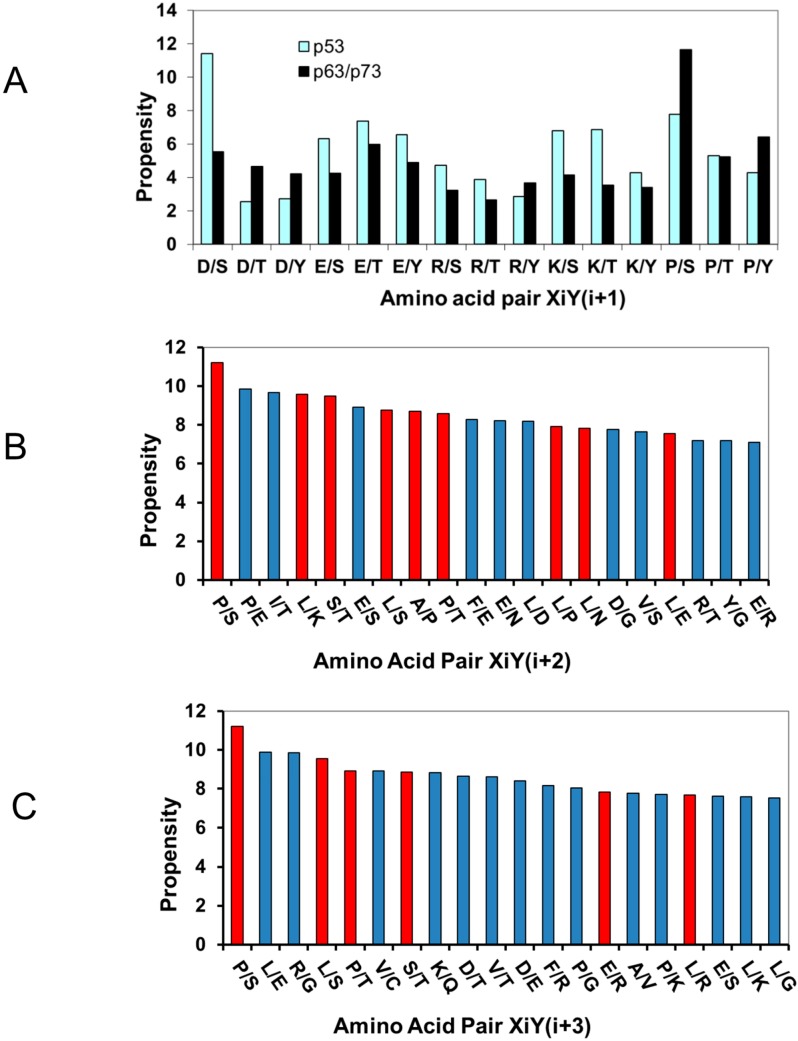
Amino acid pair propensities in p53 phosphorylation motifs follow similar trends as those of other p53 protein families, indicating evolutionary optimization of p53 phosphorylation motifs for function. (**A**) Comparison of the propensity of a charged residue (and proline) adjacent to potential phosphorylation sites shows that p53 prefers acidic residues near Ser/Thr, while p63/p73 prefers proline near Ser/Tyr. The distributions of the amino acid pair propensities DPxy among p53 pairs at the position (**B**) X_i_Y_j=i+2_ (tripeptide X × Y motif); and (**C**) pairs at the position X_i_Y_j=i+3_ (tetrapeptide X ×× Y motif). The red bar indicates pairs that are preferred for all p53, p63, and p73 proteins.

Using previous computational dipeptide propensities in p53 family proteins [77], we investigate the overall trend of a charged residue adjacent to a potential phosphorylation site in p53 family proteins. Even though p53 has no Tyr phosphorylation site, we still include Tyr for comparison. As shown in Figure 1A, among 12 possible combinations (D/S, D/T, D/Y, E/S, E/T, E/Y, R/S, R/T, R/Y, K/S, K/T, K/Y) we found that p53 has only three (D/T, D/Y, and R/Y) with lower propensity than p63/p73, and all three amino acid pairs (D/T, D/Y, and R/Y) were gradually eliminated during p53 evolution [77]. Unlike p53, Tyr phosphorylation has been reported for p73 [43]. p73 is a substrate of Tyr kinase c-Abl and the ability of c-Abl to phosphorylate p73 is markedly increased by γ-irradiation [81,82]. c-Abl recruits TP73 through interaction of its SH3 domain with the PY motif of TP73.

**Table 2:** Count of amino acid pairs in p53 phosphorylation motifs.

Amino Acid Pair	P_−1_-P(S/T)-P_+1_	P_−2_-P(S/T)-P_+2_	P_−3_-P(S/T)-P_+3_
D/S	6	2	2
P/S	6	6	8
S/Q	5	2	2
S/T	4	4	3
L/S	3	3	2
E/T	3	0	0
P/T	3	2	4
S/S	3	3	2
F/T	3	1	0
V/S	2	4	2
F/S	2	0	2
R/S	2	3	2
S/H	2	2	1
S/G	2	3	2
K/T	1	0	0
T/G	1	0	0
R/T	1	3	1
S/N	1	1	3
N/T	1	0	0
S/Y	1	0	1
S/C	1	0	0
E/S	0	3	3
Q/T	0	2	1
A/S	0	3	0
M/S	0	1	1
W/T	0	1	0
D/T	0	2	2
A/T	0	2	2
T/G	0	1	1
V/T	0	1	1
W/S	0	0	2
K/S	0	0	2
T/H	0	0	2
M/T	0	0	1
S/K	0	1	0

**Figure 2:**
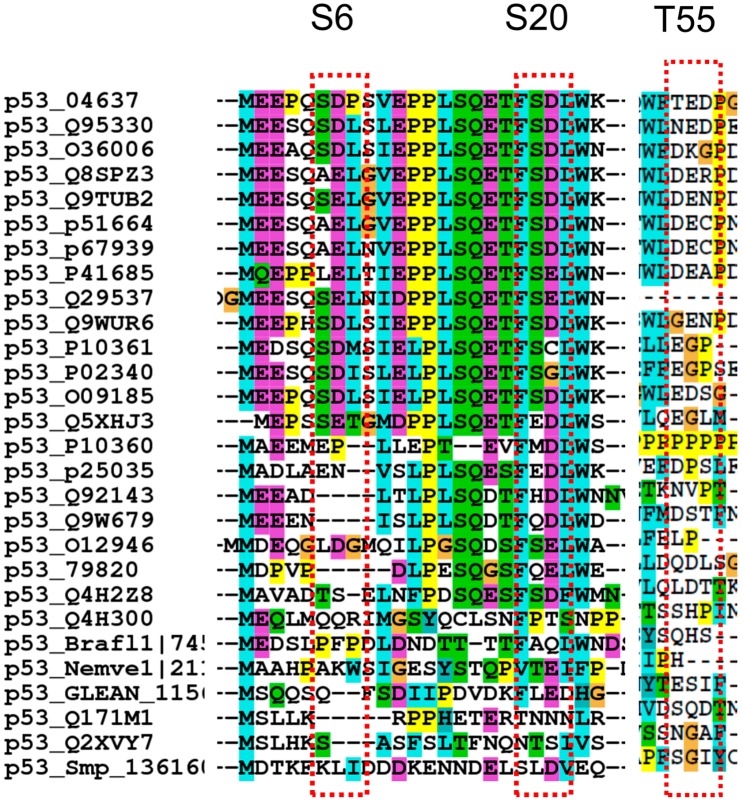
Sequence alignments near several phosphorylation sites indicate that acidic residues near phosphorylation sites are conserved in p53 proteins. The number after p53 indicates the protein access number in pubmed. See reference [77].

We conclude that the correlation of D/S and E/T is significant only for p53, reflecting an evolving requirement for p53 function (Figure 2).

#### 2.1.3. Proline Residue in P_−2_, P_−3_, P_+2_, and P_+3_ Positions in the Phosphorylation Motifs

The high occurrence of D/S dipeptides near phosphorylation sites in p53 prompted us to examine the dipeptide correlation within the phosphorylation motifs in p53 for the P_−2_-X-P(S/T)-X-P_+2_, and P_−3_-X-X-P(S/T)-X-X-P_+3_. We list the counts for observed amino acid pairs in p53 phosphorylation motifs in Table 2. For amino acids at the P_−2_ and P_+2_ positions, Pro dominates (6 P/S and 2 P/T) and Asp drops. However, if we count Glu as well, the combined contributions of Asp and Glu (2 D/S, 3 E/s, and 2 D/T) are still comparable to Pro. When we examine the distant correlations at the P_−3_ and P_+3_ positions, only proline stands out and no other amino acid has comparable frequencies.

**Figure 3:**
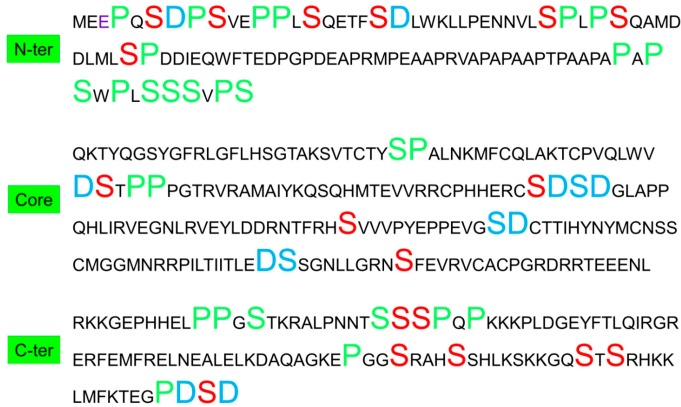
Distributions of D/S and P/S amino acids pairs in p53 indicate that the correlation of D/S (in X_i_Y_i+1_ position, Blue large fonts) and P/S (in X_i_Y_i+1_, X_i_Y_i+2_, and X_i_Y_i+3_ position, green large fonts) are mostly near phosphorylation sites. The known phosphorylation sites for Ser are in red font.

To compare the amino acid correlations in P_−2_-X-P(S/T)-X-P_+2_ and P_−3_-X-X-P(S/T)-X-X-P_+3_ positions within the phosphorylation motifs, we calculated the amino acid correlations for all amino acids in X_i_Y_i+2_ and X_i_Y_i+3_ pairs in p53 family proteins. As can be seen in Figure 1B,C, P/S has the highest propensity to appear in both X_i_Y_i+2_ and X_i_Y_i+3_ positions in p53. The high P/S correlations in phosphorylation motifs follow the overall amino acid correlations in the p53 protein. As can be seen in Figure 3, the P/S amino acid pairs predominately locate in *N*- and *C*-terminals and mostly near phosphorylation sites. Therefore, it is plausible to assume that the high propensities of P/S to correlate at the X_i_Y_i+2_ and X_i_Y_i+3_ positions are mostly driven by specific requirements for p53 phosphorylation.

### 2.2. Cancer Mutations in p53 Phosphorylation Motif

#### 2.2.1. D/S (E/T) Pairs Decrease while P/S Pair Increase Mutation Counts in p53 Phosphorylation Motif

Phosphorylation could be perturbed by mutations not only directly at the phosphorylation site, but also near mutations within the motif. These mutations could change p53 kinase specificity or modify the biological responses following phosphorylation. For example, the Q16L and Q100K mutations would abolish the SQ motif, which is needed for ATM and ATR recognition. Mutation of Arg at P_−2_ positions to S183, T211, S215, and S269 might turn off Aurora-B recognition. It is known that phosphorylation of nearby sites (like S6/S9 and S33/S37) are interdependent [83,84], which is equivalent to perturbations near the phosphorylation site.

**Figure 4:**
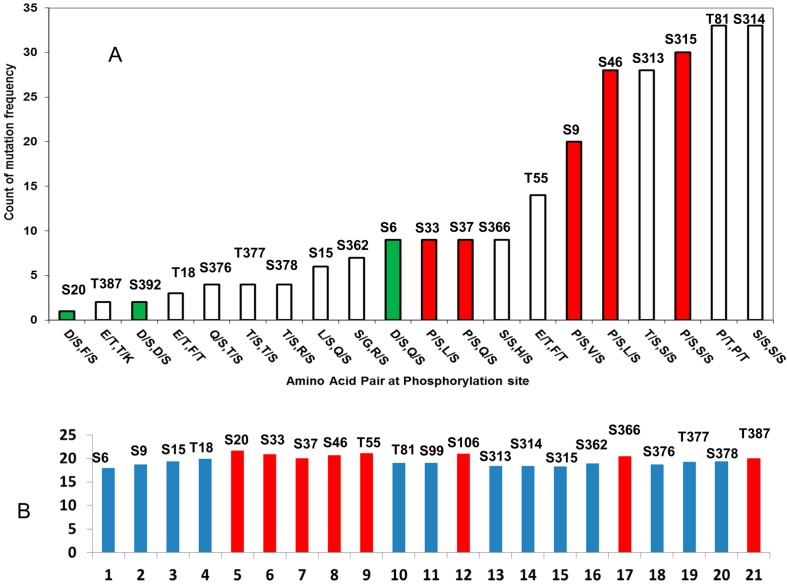
p53 phosphorylation motifs can be characterized by amino acids adjacent to the phosphorylation motif and the propensity of structural disorder of the seven residue phosphorylation motif. (**A**) Phosphorylation motifs with D/S pattern (green bar) have fewer mutations than the motif with the P/S pair (red bar); (**B**) Phosphorylation motifs are structurally disordered (blue bar), some motifs, which are less disordered (red bar), have more secondary structure characteristics.

We probed the p53 mutation database (IARC p53 mutation database Release R16) to obtain the frequencies of the mutation within the phosphorylation site motifs. In Table 3, we list the number of observed mutations within the seven residue phosphorylation motifs. Since the core domain group has a high number of mutations, we put the core domain in a separate category, and combine the *N*- and *C*-terminal sites. Several observations can be made from the Table 3. (1) Even though many phosphosrylation sites do not have mutations (S9, T18, S20, T55, S362, S377, S378, T387), mutations still occur within all phosphorylation motifs; (2) Mutation counts are not randomly distributed among the seven positions in the phosphorylation motifs; the P_−1_P_0_ positions have the lowest counts, and the P_+1_P_+2_P_+3_ positions have more mutations than at P_−1_P_−2_P_−3_ positions; (3) Mutational counts are higher when there is proline before or after the phosphorylation site (P/S and P/T), while the phosphorylation motifs with the least mutations often have negatively charged amino acids before or after the phosphorylation site (D/S and E/T, also see Figure 4A); (4) For *N*- and *C*-terminal regions, the motifs having consensus phosphorylation sites have a higher average mutational rate (16.7/motif) than those that do not follow the consensus phosphorylation sequences (11.7/motif), suggesting that phosphorylation specificities might be sensitive to mutations within the motifs.

**Table 3:** Mutations observed in the phosphorylation site motifs of p53 (Bold fonts are for motifs not following consensus sequence).

pS/pT	P_−3_	P_−2_	P_−1_	P_0_	P *_+_*_1_	P *_+_*_2_	P *_+_*_3_	Total	Amino Acid Pair
*N*- and *C*-Terminus Domains									
S6	0	3	1	2	1	2	0	9	D/S, Q/S
S9	2	1	2	0	1	11	2	19	P/S, V/S
S15	2	1	0	1	1	1	0	6	L/S, Q/S
**T18**	**1**	**1**	**1**	**0**	**0**	**0**	**0**	**3**	**E/T, F/T**
S20	1	0	0	0	0	0	0	1	D/S, F/S
S33	1	2	0	1	1	3	1	9	P/S, L/S
S37	1	3	1	2	0	2	0	9	P/S, Q/S
S46	1	4	1	5	8	1	8	28	P/S, L/S
**T55**	**1**	**4**	**2**	**0**	**5**	**0**	**2**	**14**	**E/T, F/T**
T81	3	4	2	2	9	3	10	33	P/T, P/T
S99	6	3	6	4	1	4	7	31	P/S, Q/S
**S106**	**0**	**3**	**11**	**8**	**5**	**3**	**6**	**36**	**S/G, S/Y**
**S313**	**4**	**6**	**8**	**4**	**1**	**3**	**2**	**28**	**T/S, S/S**
S314	6	8	4	1	3	2	9	33	S/S, S/S
S315	8	4	1	3	2	9	3	30	P/S, S/S
**S362**	**0**	**1**	**0**	**0**	**1**	**3**	**2**	**7**	**S/G, R/S**
**S366**	**2**	**3**	**2**	**2**	**0**	**0**	**0**	**9**	**S/S, H/S**
S376	0	0	0	2	0	0	2	4	Q/S, T/S
**T377**	**0**	**0**	**2**	**0**	**0**	**2**	**0**	**4**	**S/S, S/S**
S378	0	2	0	0	2	0	0	4	T/S, R/S
**T387**	**0**	**1**	**0**	**0**	**0**	**1**	**0**	**2**	**E/T, T/K**
**S392**	**1**	**0**	**0**	**1**	**0**			**2**	**D/S, D/S**
Sum (*N*- and *C*-terminus)	41	54	44	38	41	50	54		
**Core Domain**									
S149	14	32	16	16	14	190	141	423	D/S, T/S
T150	32	16	16	14	190	141	36	445	T/S, P/T
T155	141	36	85	79	85	228	247	901	S/G, R/S
S183	18	88	13	6	39	16	15	195	S/D, S/C
T211	39	15	14	30	16	81	87	282	T/N, F/T
S215	16	81	87	86	94	16	45	425	S/H, S/V
S269	187	57	17	20	102	54	169	606	N/S, F/S
Sum (core domain)	447	325	248	251	540	726	740		

Based on IARC p53 mutation database Release R16, which contains 29573 somatic mutations in sporadic cancers reported [85].

Apparently, the phosphorylation motifs with proline near phosphorylation sites and with consensus phosphorylation sequences are more sensitive to mutations. It is interesting to examine why phosphorylation motifs with negatively charged amino acids near the phosphorylation site have the lowest mutation rate. Charged residues, especially acidic residues, are more likely to be involved in structurally disordered regions. Among the top three residues with the highest propensity to be in disordered regions (Gly, Asp, and Pro), Asp ranks the second [78]. We then study the disorder propensities of p53 phosphorylation motif.

#### 2.2.2. Mutations Decrease the Propensity of Disorder in p53 Phosphorylation Motifs

**Figure 5:**
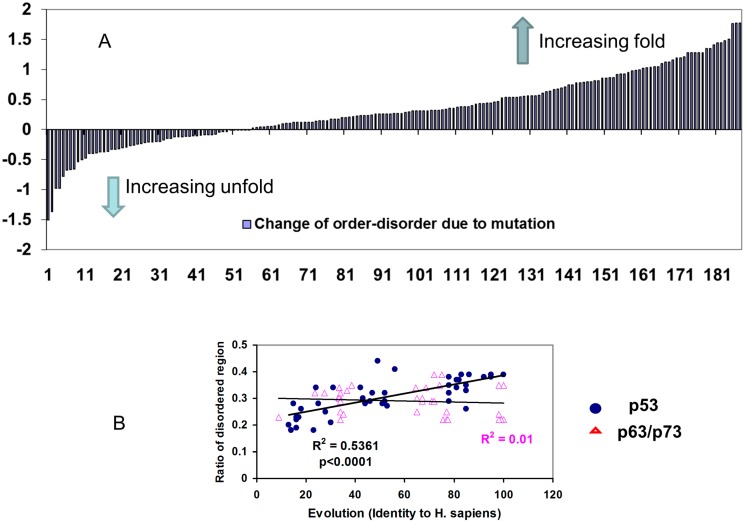
Evolution optimized p53 to have more structural disorder and p53 mutations tend to reverse the trend. (**A**) The majority of p53 mutations in phosphorylation motifs increase folding (decrease disorder) propensities of the seven residue phosphorylation motifs; (**B**) The ratio of disordered region in p53 increases with p53 evolution, but there is no such correlation for p63/p73.

Most of p53 phosphorylation sites are natively disordered, as is the general case in phosphorylation sites [86,87]. To clarify the effects of p53 cancer mutations on the local structures near the phosphorylation site, we calculated the fold-unfold index of the seven residue phosphorylation motifs in the *N*- and *C*-terminals before and after mutations. An increase of fold-unfold index indicated that mutations cause the phosphorylation motif to have a more folded structure, while the decrease of fold-unfold means that he motif is more disordered after the mutation. As indicated in Figure 5A, we found that most mutations increase the folded structures of the phosphorylation motif (132 counts) and less than one third (55 counts) increase the disordered nature of the phosphorylation motifs. Notably, many phosphorylation motifs have the fold-unfold index right below the cutoff limit (20.4) and disordered (Figure 4B). However, cancer mutations increased the fold-unfold index and the phosphorylation motifs can become structured with the mutation present. For example, Q16L increased the index of the Ser15 motif (PPLS15QET) from 19.4 to 20.4, P80S mutation increased the index of the Thr81 motif (AAPT81PAA) from 19.1 to 20.4, and the G389W mutation boosts the index of T387 motif (MFKT387EGP) from 20.0 to 21.5. These results indicated that beside the effect of changing the phosphorylation specificity, the change in the flexibility of the phosphorylation motif could be one of the common mechanisms of mutational effects on p53 phosphorylation.

It is known that human p53 is less stable than p53 proteins in other species [88,89]. To further investigate the evolutionary divergence of p53 structure, we calculated the fold-unfold index for all known p53 protein families. As indicated in Figure 5B, we found that the p53 protein is gaining disorder during evolution, while the fold-unfold index of p63/p73 protein has no correlation with evolution. Since most of the disordered segments are in the *N*- and *C*-terminal regions, it appears that the phosphorylation motifs in these regions have become increasingly disordered as well.

## 3. Discussion

While overly simplified, the differences between the p53 and p63/p73 phosphorylation motifs can be characterized by preference for two types of dipeptide correlation: D/S and P/S pairs. Our bioinformatics modeling suggests that p53 prefers combining Asp and Ser (D/S pair); while p63/p73 have an opposite preference for the P/S pair and less of the D/S pair. A study to predict potential phosphorylation sites in ∆Np63α also revealed the preference of P/S pair in phosphorylation motifs of p63, and it was found that among 20 predicted serine phosphorylation residues, eight are P/S pair and only two are D/S pair [90]. Since p63/p73 are evolutionary older than p53, we may argue that p53 adapted the D/S (or E/T) combination and reduced P/S (or P/T) association to make the phosphorylation sites in p53 less prone to mutations. At the same time, the intrinsically disordered nature of most p53 phosphorylation sites makes them more adaptive, providing strong resistance to deleterious mutations within p53 phosphorylation motif.

### 3.1. Why Is P/S (or P/T) Association More Vulnerable to Cancer Mutation?

There are three possible explanations for our observations. (i) From structural point of view, kinases use a proline near the phosphorylation site to precisely align the substrate site to the catalytic pocket. Therefore, mutant substrate binding is easier to be affected by mutation; (ii) p53 needs to integrate dozens of phosphorylation signals in response to various types of cell stress. p53 often binds different kinases with the same phosphorylation motif. Since these kinases may not share sequence specificity, the P/S pair may restrict p53’s flexibility and is sensitive to perturbation; (iii) p53 needs to be distinguished from p63/p73. p53/p63/p73 interfere with one another [91]. Therefore, if p63/p73 have high propensity to have Pro/Ser (or Pro/The) associations within the phosphorylation motif, p53 may have to adopt a different pattern to avoid interference with p63/p73 before/after phosphorylation triggered responses. Since DNA binding and oligomerization domains have to be conserved, the post-translational modifications pattern can be effective ways to drift away from p63/p73. The absence of Tyr phosphorylation in p53 can be looked at from this angle as well.

### 3.2. Acidic Amino Acids Adjacent to Phosphorylation Sites in p53 Protein Families Can Provide Phosphorylation Redundancy

It has been puzzling that the phosphorylation sites themselves are rarely mutated in cancer [92]. Individual p53 phosphorylation events could be redundant [93]. Extensive studies have shown that no single PTM appears to be essential for TP53-mediated tumor suppression [67]. Still, the tumor mutations at these sites and flanking residues are significant (Table 3 and Figure 4A), suggesting that modifications of these phosphorylation motifs might contribute to TP53 mediated tumor suppression [67]. The redundancy may reflect the preference of p53 for D/S and E/T pairs in their phosphorylation sites. Such charge redundancy can also be seen in several other phosphorylation sites like S20 and T55 not conserved within p53, often replaced by either D or E in non-homo-sapiens species (Figure 2).

The charge redundancy could have achieved incremental smooth transition triggered by phosphorylation, in addition to evolutionary pressure to fend off mutations. The Asp/Ser (and Glu/Thr) combinations in phosphorylation sites decrease the variability of electrostatic interactions, limiting the extent of binding energy change upon phosphorylation. Since the change of transcriptional factor binding energy is correlated with transcriptional response [94], charge redundancy might be a way to control p53 transcriptional response.

From the electrostatic interaction point of view, the D/S combination reduces the extent of charge change when adding two more negative charges from phosphorylation of Ser. With a negative charge already near the site, the change of charge density is 200% (from −1 to −3 or from −3 to −1 in case of dephosphorylation). In comparison, the change of charge density would be steeper with a neutral residue or a positively charge residue adjacent to the phosphorylation site (switch between 0 and −2, or between +1 and −1). The smoother change of electrostatic interactions upon phosphorylation would translate into smoother change of binding energy involving the phosphorylation site with other proteins. The combination of negative charge with phosphorylation would make a mutation in the phosphorylation site less deleterious, since the motif already has a negative charge and may still have certain electrostatic interactions with the other binding partner.

The existing experimental data support the hypothesis that D/S and E/T pairing would provide a smoother transition (Table 4). The T14 phosphorylation site in p73 (PDGGT_14_TFEHLW) can be comparable to T18 in p53 (S_15_QET_18_FS_20_DLW), with similar sequence and function [51]. However, the p53 *N*-terminal region phosphorylation sites around S15, T18, and S20 are surrounded by Asp and Glu residues, while the p73 has charged residues relatively away from the phosphorylation site T14. Therefore, we expect that T14 phosphorylation in p73 would increase the binding affinity to a larger extent than phosphorylation of T18 in p53. As shown in Table 4, generally, phosphorylation of p53 increases the binding affinity with the TAZ1 or TAZ2 domains of p300/CBP by two to seven folds. With more comparable experimental setting, the phosphorylation of the T14 of p73 increases the binding affinity by 10-fold, significantly higher than that for p53 [51,95].

**Table 4:** Experimental dissociation constants of p53/p73 *N*-terminal with CBP/p300.

Peptides	Kd μm				Ref.
	P300/TAZ1	P300/TAZ2	CBP/TAZ1	CBP/TAZ2	
P73(10–40) wt	39	4.5			[51]
P73(10–40) pT14	4.6	0.47			
P53(1–57) wt		0.77			[95]
P53(1–57) pT18		0.11			
P53(10–57) wt		0.88			
P53(10–57) pS15pS20		0.21			
P53(13–61) wt			0.9	0.026	[96]
P53(13–57) pT18			0.5	0.05	
P53(13–57) pS15pT18pT20			0.07	0.08	
P53(1–39) wt		0.43 (7.15) *			[97] *
P53(1–39) pS15		0.05 (1.83) *			
P53(1–39) pT18		0.05 (1.05) *			
P53(1–39) pS15pT18		0.05 (1.74) *			
**Peptides**	**Kd μm**				**Ref.**
	**TFB1**	**P62 (wt)**	**P62 (K18E)**		
P53(25–65) wt	0.39	3.18	3.63		[98]
P53(25–65) pS46	0.15	0.52			
P53(25–65) pT55	0.16	0.46			
P53(25–65) pS46pT55	0.07	0.10	4.44		

* Salt concentration. 50 mM and 200 mM (in bracket).

P53 may use multiple phosphorylation events to gradually increase its binding affinity with other proteins to different extents. As can be seen in Table 4, double phosphorylation of S15 and T18 for p53(1–39) essentially does not change the binding with the p300/TAZ1 domain [97]; while the triple phosphorylation of p53(13–57) weakens the interaction with CBP/TAZ2 domain by more than 10 fold [96].

The electrostatic effect of p53 phosphorylation can also be illustrated by p53 interaction with the TFB1/p62 subunit of transcription factor II H (TFIIH) [98]. The highly negatively charged p53 TAD 2 domain (MLS_46_PDDQWFT_55_EDP) folds into an α-helix and binds to the positively charged region on TFB1/p62. Phosphorylation of either S46 or T55 increases the affinity and double phosphorylation of S46 and T55 further reinforcing the p53 interaction with TF1B1/p62 (Table 4) [98]. It is interesting to note that when one of the salt bridges is disabled by the K18E mutation on p62, p53 still has weak interaction with the K18E p62 mutant, while double phosphorylated p53 becomes more repulsive to p62 (Table 4).

### 3.3. The Natively Disordered Nature of p53 Phosphorylation Motifs: Vulnerable or Resistant to Mutations?

The structures of p53/63/p73 contain an *N*-terminal transactivation domain (TA), a DNA-binding core domain, a *C*-terminal tetramerization and a regulatory domain. In addition, p63 and p73 also have a sterile alpha motif domain (SAM) at the end of the *C*-termini. Most of the *N*-terminal TA domain and *C*-terminal regulatory domain are highly flexible and have no well-defined structure. Therefore, p53 can also be classified as a typical intrinsically unstructured protein [99,100], which is frequently observed for gene regulating proteins [87,101]. The advantages of the conformational flexibility of the disordered region, coupled with extensive posttranslational modifications, make the p53 amenable for regulation for its complex cellular tasks [102,103].

It was suggested that natively disordered proteins are more likely to be associated with cancer [101]. p53 is an intrinsically unstructured protein and is the most mutated protein in cancer. However, the phosphorylation motifs in p53, even with considerable mutations, are “rare” comparing with its core domain. Therefore one may ask if the natively disordered nature of p53 phosphorylation site is vulnerable or resistant to cancer-related mutations. It is advantageous to adapt the p53 order/disorder transition by phosphorylation for integrating stress signals and providing graded responses [103]. Meantime, with the order/disorder transition, a rich conformational ensemble and low barriers may buffer mutational perturbations [102]. As a result, it could make p53 less vulnerable to mutations. Should the phosphorylation motifs of p53 be more rigid, they could have had more oncogenic mutations in their phosphorylation motifs. Thus, the fact that many natively disordered proteins are related to cancer could also be due to these proteins tending to integrate cell signals and pathways. The nature of multi-functional proteins may be associated with cancer because of propagation of mutational effects.

### 3.4. Implications of Targeting p53 Mutants in Cancer Therapy

Gene therapy to restore p53 function is among the clinical approaches currently under investigation [61]. For example, apoptosis induced by adenovirus-mediated *p53* gene transfer in human glioma correlates with site-specific phosphorylation [103,104], suggesting that it is possible to deliver engineered p53 with acidic amino acids adjacent to potential phosphorylation sites. Based on our study, these types of engineered p53 may compensate phosphorylation deficit and still leave the phosphorylation site unchanged. Such approaches could provide an alternative to phosphorylation-mimicking mutations, which can change the relative susceptibility of cells to the harmful effect of ionizing radiation [33]. It is known that p53 phosphorylation plays a role in regulation of the neoplastic proliferation of cells in radio/chemotherapy. Restoration of mutant p53 to wild-type p53 is also a promising cancer therapy, and several drugs are under development aiming to stabilize p53 mutants [61]. While stabilizing the core domain has proven an effective strategy, such approaches would need to be modified to target p53 phosphorylation-site related mutations.

## 4. Materials and Methods

### 4.1. Amino Acid Pair Correlation and Propensities

For each sequence in a protein family, we count the two amino acids (X and Y) in the positions of X_i_Y_j=i+1_, X_i_Y_j=i+2_, and X_i_Y_j=i+3_, which are equivalent to the dipeptide XY, tripeptide X × Y, and tetrepetide X ×× Y motifs, respectively. Then we calculate the propensity of amino acid pair association by normalizing the frequencies of the overall counts of individual amino acid in the p53, p63, or p73 families:

P_XiYj_ = 100 × N/(N + N) (1)

where P_XiYj_ is the propensity of amino acid pair association in X_i_Y_j=i+1_, X_i_Y_j=i+2_, and X_i_Y_j=i+3_ positions, respectively. N_XiYj_ is the total number amino acid pair counts in X_i_Y_j=i+1_, X_i_Y_j=i+2_, and X_i_Y_j=i+3_ positions in all sequences in each family, respectively. The N_X_ and N_Y_ are total numbers of amino acid type (X and Y) in all sequences in each p53, p63, or p73 families [77]. Thus, an amino acid pair would have higher propensity if it has higher frequency in a sequence and highly conserved within a family. The above P_XiYj_ is similar to that used by Vonderviszt and Simon [104], who used N_XiYj_/(N_XY_ × P_X_ × P_Y_) to measure the dipeptide propensity (N_XY_ is the total number of all dipeptides, P_X_ and P_Y_ are the relative abundances of amino acid types X and Y). P_XiYj_ in equation 1 changes more smoothly with the variation of N_X_ and N_Y_ in the calculation of the dipeptide propensities across evolution [77].

We calculate the degenerate dipeptide pair correlation propensity DP_XiYj_ by adding P_XiYj_ and P_YiXj_ for non-diagonal elements in the correlation matrix.

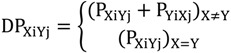
(2)


Thus, we do not distinguish between XY and YX and label such amino acid combinations X/Y, and the propensity X/Y is obtained from the combined XY and YX counts.

### 4.2. Disorder Propensities of Phosphorylation Motif and Proteins in the p53/p63/p73 Family

Disorder propensities were calculated using the webserver FoldUnfold [78]. The average frames 7 was selected for seven residue phosphorylation motif, and the frames 11 was used in predictions of entire protein sequence. For each phosphorylation motif, we first calculate the disorder propensities for wt p53, then the disorder propensities were recalculated to reflect the mutations within the motif.

We use ClustalX 2.0 [105] to align the sequences of p53 family proteins [77]. We use sequence identities with human p53 as the measure of evolutionary distances. For the p63/p73 family, we use the averaged sequence identities with human p63 and p73.

## 5. Conclusions

The existence of almost thirty phosphorylation sites in p53 poses a considerable evolutionary pressure on p53 to selectively bind to specific kinase with the right phosphorylation motif to respond to correct phosphorylation signals. What are the sequence and structural features in p53 phosphorylation motifs? Why and how p53 phosphorylation sites are rarely mutated in human cancer? In our study, we focused on the phosphorylation sites and nearby flanking residues and computationally examined the consensus phosphorylation motif pattern, amino acid correlations within the phosphorylation motifs, the propensity of structural disorder of the phosphorylation motifs, and cancer-related mutations observed within the phosphorylation motifs. Among the many factors potentially contributing to the safeguarding mechanism against mutations in p53, we found two patterns that appear unique and correlated with p53 mutations. p53 proteins have high propensities for acidic amino acids adjacent to potential phosphorylation sites. The negative charge near a phosphorylation site might make a mutation of the phosphorylation site less deleterious, since the motif already has a negative charge and may still have certain electrostatic interactions with other binding partners. The above conclusion is consistent with experimental observations. p53 phosphorylation motifs are mostly disordered. Even though cancer-related mutations largely decrease the disorder propensity of the phosphorylation motifs, the disordered nature of phosphorylation motifs might still help to compensate for mutational effects on p53 phosphorylation. Our computational results suggest that engineered acidic amino acids adjacent to potential phosphorylation sites could be a potential strategy in *p53* gene therapy.
